# Diagnostic biomarkers for non-traumatic osteonecrosis of the femoral head: a systematic review and pathophysiological cascade network

**DOI:** 10.1302/2046-3758.159.BJR-2026-0020.R1

**Published:** 2026-09-21

**Authors:** Yawei Dong, Jigao Sun, Shuwen Li, Jiawen Zhang, Yan Yan, Taixian Li, Yan Jia

**Affiliations:** 1 Joint Surgery Department III, Wangjing Hospital of China Academy of Chinese Medical Sciences, Beijing, China; 2 Orthopedics Department, Dongfang Hospital Beijing University of Chinese Medicine, Beijing, China; 3 Dongguan Hospital of Guangzhou University of Chinese Medicine, Dongguan Traditional Chinese Medicine Hospital, Dongguan, China; 4 Traumatic Joint Surgery Department, Third Affiliated Hospital of Beijing University of Chinese Medicine, Beijing, China; 5 School of Orthopedics and Traumatology, Chongqing University of Chinese Medicine, Chongqing, China; 6 Pain Department, Shanxi Bethune Hospital, Shanxi Academy of Medical Sciences, Third Hospital of Shanxi Medical University, Tongji Shanxi Hospital, Taiyuan, China

**Keywords:** Non-traumatic osteonecrosis of the femoral head, Biomarkers, Diagnostic prediction, Progression prediction, biomarker(s), Avascular Necrosis Of the Femoral Head, RNAs, blood, pathogenesis, autophagy, clinical diagnosis, femoral head, microRNAs, Inflammation

## Abstract

**Aims:**

Many studies have focused on identifying specific biomarkers for the early diagnosis of non-traumatic osteonecrosis of the femoral head (NONFH). This systematic review aims to summarize biomarkers associated with the diagnosis and progression prediction of NONFH, thereby providing a molecular level foundation for clinical diagnosis and targeted treatment.

**Methods:**

Following the PRISMA guidelines for systematic reviews, we comprehensively searched original English-language articles published between January 2015 and December 2025 in the Web of Science and PubMed databases. Studies were selected based on the PICOS framework. Extracted data included study design, methodologies, sample sources, identified biomarkers, predictive performance, and the potential pathological pathways involved.

**Results:**

Overall, 15 studies were included, reporting a total of 52 biomarkers, all derived from blood samples. Biomarkers from eight studies were used to reflect NONFH progression, while those from seven studies were using for diagnostic prediction purposes. All biomarkers demonstrated favourable predictive performance, with six studies providing external validation. The predictive model comprising ICAM1, NR3C1, IKBKB, and CD4 showed promising performance, with area under the receiver operating characteristic curve (AUC) values each reaching 1.00. Based on their core functions, the biomarkers were categorized into seven functional groups: immune-inflammatory activation and response, signal transduction and regulation, molecular metabolism and energy homeostasis, vascular function and coagulation, oxidative stress, pyroptosis and autophagy, and RNA regulatory.

**Conclusion:**

Biomarkers for NONFH exhibit considerable potential for early diagnosis and disease progression prediction in the Chinese population. The pathogenesis of NONFH involves a multifaceted pathological process in which various components interact and synergistically exacerbate the condition. This indicates that future therapeutic strategies should adopt a multitarget, multipathway synergistic intervention approach.

Cite this article: *Bone Joint Res* 2026;15(9):1159–1175.

## Article focus

- The potential of specific biomarkers for non-traumatic osteonecrosis of the femoral head (NONFH) warrants further investigation.
- The primary aim of this systematic review is to integrate the predictive performance and pathophysiological network of existing biomarkers associated with NONFH, including proteins, metabolites, genes, and RNAs.

## Key messages

- This study suggests that NONFH biomarkers are involved in a pathophysiological cascade which may be driven primarily by inflammatory responses.
- These biomarkers show promising performance in the early diagnosis of NONFH or in predicting disease progression in the Chinese population, indicating their potential as novel intervention targets for NONFH.

## Strengths and limitations

- The integrated findings of this systematic review indicate that the pathological process of NONFH constitutes a synergistic and escalating cascade network initiated by ‘immune activation-chronic inflammation’.
- Future therapeutic strategies should adopt multitarget, multipathway combinatorial interventions.
- While studies on biomarkers for NONFH have been widely reported, robust systematic evidence supporting the clinical validity of most proposed markers remains limited.

## Introduction

Non-traumatic osteonecrosis of the femoral head (NONFH) is a refractory bone disease that affects people of all ages, from children to the elderly. It is more common among adults aged between 20 and 50 years.^1^ NONFH progresses so rapidly that it is almost impossible to prevent disability. One study has estimated that femoral head collapse occurs in approximately 67% of asymptomatic patients and 85% of symptomatic patients.^2^ Without intervention, 60% to 80% of early-stage patients will deteriorate into femoral head collapse and even secondary hip osteoarthritis, forcing them to undergo total hip arthroplasty in order to regain hip joint function.^3^

The pathogenesis of NONFH is complex with multiple inducing factors. First and foremost is glucocorticoid, the long-term and high-dose application of which may cause microvascular circulation disorders, thereby activating bone cell necrosis. In China, steroid-induced osteonecrosis of the femoral head (SONFH) has become the most common type.^4^ The role of alcohol should not be overlooked either: alcohol-induced lipid metabolism disorder may be the core link, making men with a long history of drinking or alcohol abuse more likely to be diagnosed with alcohol-induced osteonecrosis of the femoral head (AIONFH) in middle age.^5^ In addition, decompression sickness and sickle cell disease have also been added to the list of aetiologies in recent years.^6^ However, there are still many patients whose aetiology remains unknown.

There is an inherent delay in the diagnosis of NONFH when relying on imaging methods; consequently, the development of accurate and more convenient predictive approaches has emerged as a key research priority. Susceptibility gene polymorphism once served as a foothold, providing preliminary clues to help explain individual differences in NONFH.^7,8^ The rapid development of multiomics technologies has offered a research framework based on systematic networks for NONFH. Among these, transcriptomics can reveal differences in gene expression profiles at different stages of the disease and accurately capture the activation status of key pathways;^9^ proteomics can directly detect the expression levels of proteins in the lesion microenvironment and identify core molecules that drive the pathological process.^10,11^ Through multidimensional analysis of clinical samples, a growing number of researchers have successfully identified a batch of potential biomarkers closely associated with the early diagnosis, assessment of disease severity, and prognostic outcome of NONFH, including differentially expressed proteins, characteristic metabolites, and circulating microRNAs.^12^

Based on this, this review systematically summarizes the current research status of biomarkers for NONFH, delineating their discovery pipelines, clinical values, and validation progress. It aims to gain a deeper insight into the key pathogenic triggers at the molecular level, and lay a foundation for precise diagnosis and treatment strategies.

## Methods

### Search strategy

This systematic review was conducted in accordance with the list of preferred reporting items for systematic reviews and meta-analyses (PRISMA 2020), and has been registered in PROSPERO (CRD420261359314).^13^ A systematic review was conducted on the published articles analyzing the biomarker characteristics of NONFH. All relevant articles were retrieved from databases represented by PubMed and Web of Science. The time limit was the decade spanning 2015 to 2025, and the search terms included a combination of standardized index terms and plain language: “osteonecrosis of the Femoral Head”, “Avascular Necrosis Of the Femoral Head”, and “Avascular Necrosis of Femur”, “Head”, “biomarker”, and “biomarkers”, corresponding search strategies should be formulated for different databases.

NONFH-related terms included nontraumatic osteonecrosis of the femoral head, avascular necrosis of femoral head, avascular necrosis of femur, ischemic necrosis of femoral head, aseptic necrosis of femur head, and femur head necroses.

Biomarker terms included: biomarker(s), biological marker(s), biologic marker(s), clinical marker(s), immune marker(s), immunologic marker(s), laboratory marker(s), serum marker(s), viral marker(s), biochemical marker(s).

### Inclusion and exclusion criteria

This systematic review is limited to observational studies with control groups, including cohort studies and case-control studies. It also complies with the PICOS framework.^14^

Population (P): NONFH in adults with a definite diagnosis (steroid, alcoholic, idiopathic).

Index test/Exposure (I): non-imaging biomarkers, including but not limited to protein, enzyme, metabolite, micro RNA (miRNA)/long non-coding RNA (lncRNA), genes in blood/urine/synovial fluid.

Comparator (C): healthy controls or non-NONFH orthopaedic disease controls; different disease courses/stages (such as ARCO I-II vs III-IV); different outcome groups (collapsed vs non-collapsed; successful hip preservation vs failed).

Outcomes (O): expression level; predictive performance (AUC, sensitivity, specificity); the association with disease stage, necrotic area, clinical phenotype, etc.

Study design (S): clinical observational studies (cohort, case-control, cross-sectional); nested case-control or clinical sample database analysis.

In addition, literature was excluded according to the following criteria: therapeutic targets; non-English articles; review, meta-analysis and/or systematic review, case report; the full text was not available; and/or the original text could not be obtained or had been retracted.

### Data extraction

Two researchers (YD, JS) independently screened the titles/abstracts and full texts, using a pre-designed screening table to record the reasons for exclusion. From the included studies, the following data were extracted, summarized and analyzed: first author, year, country, study design, study subjects, sample size, sample type, research methods, biomarker type, biomarker, receiver operating characteristic (ROC) curve index, and possible involvement of the biomarker in NONFH pathology.

### Risk of bias assessment

The QUADAS-2 tool was applied to assess the risk of bias and applicability of each diagnostic accuracy estimate,^15^ with two reviewers (YD, JS) independently evaluating each appraisal; any disagreements were resolved through discussion or consultation with a third reviewer (TL). For each domain, judgements were made regarding the risk of bias (low, unclear, high) and applicability concerns.

### Data synthesis

Due to the lack of necessary data for meta-analysis, a qualitative integration of the included literature was conducted according to biomarker category, aetiological type, and clinical use (diagnosis/prognosis prediction). To enhance the confidence of the results, a Grading of Recommendations Assessment, Development, and Evaluation (GRADE) assessment was conducted for the biomarkers.

### Function enrichment analysis

Targets interaction network was constructed using the STRING database (STRING Consortium, Swiss Institute of Bioinformatics, Switzerland) and subsequently optimized using Cytoscape software (v. 3.8.2). To elucidate the biological functions associated with biomarkers, Gene Ontology (GO) annotation and Kyoto Encyclopedia of Genes and Genomes (KEGG) pathway enrichment analyses were performed. These analyses were conducted using the Database for Annotation, Visualization and Integrated Discovery (DAVID) v. 6.8 (National Institutes of Health, USA) and the KEGG database (Kanehisa Laboratories, Japan). The GO analysis encompassed three categories: biological process (BP), cellular component (CC), and molecular function (MF).

## Results

### Search results

**Fig. 1:**
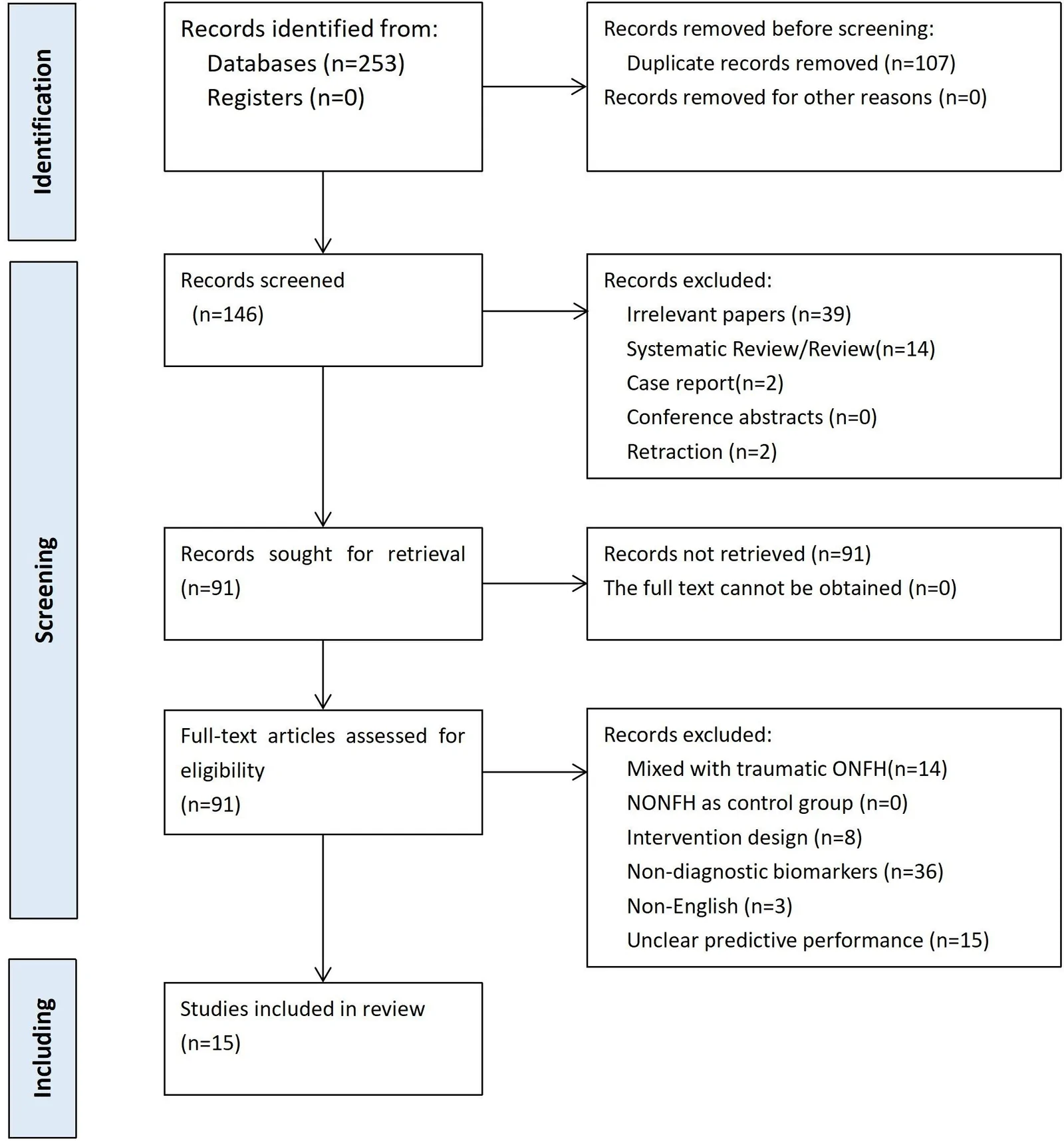
PRISMA flowchart of the systematic review.

**Fig. 2:**
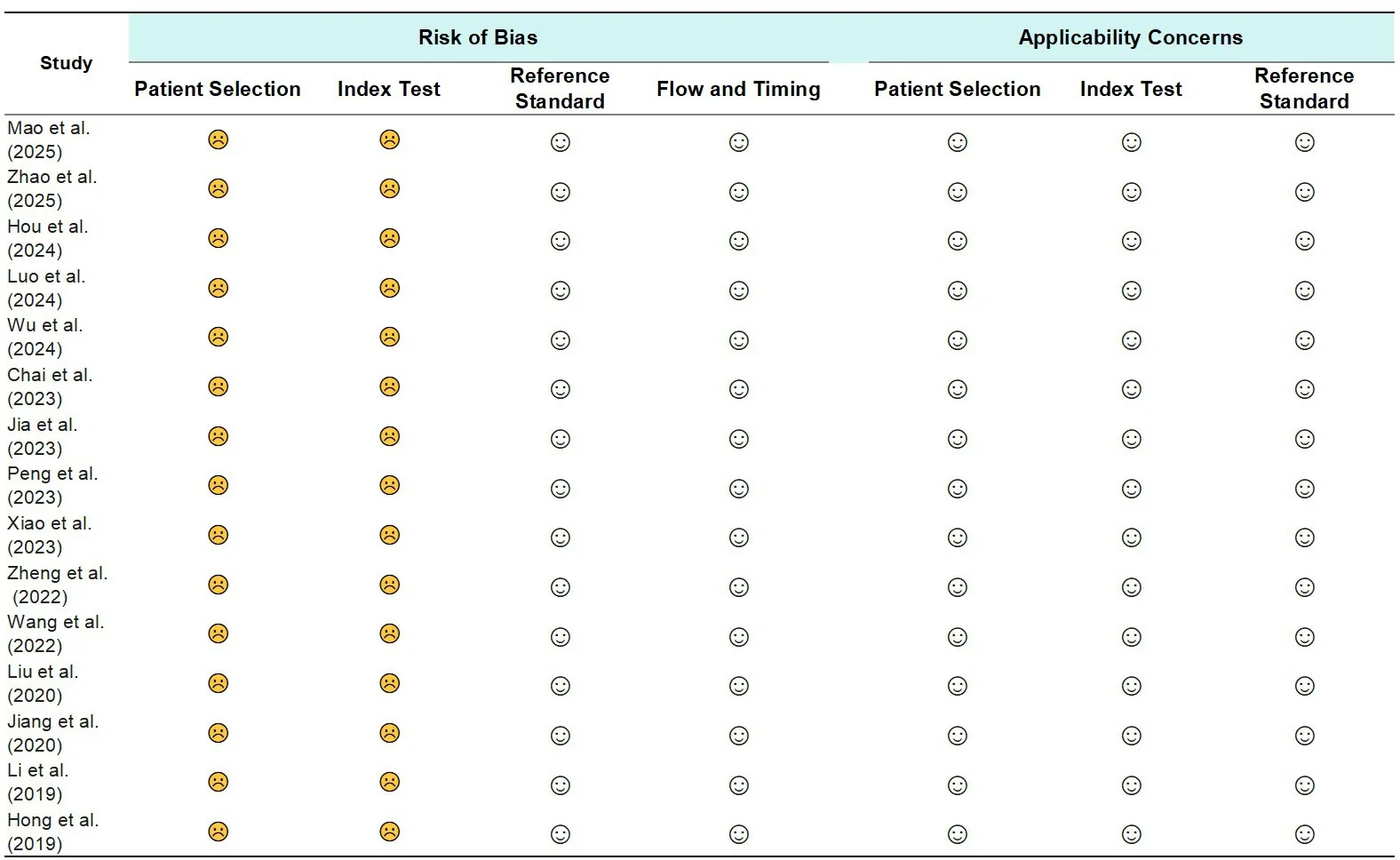
Risk of bias by QUADAS-2 tool.

The selection procedure of included articles and risk of bias analysis are shown in Figure 1 and Figure 2. A total of 253 relevant studies were identified through the systematic search. After removing duplicates, 146 studies remained. Subsequent screening of titles and abstracts based on the inclusion and exclusion criteria led to the exclusion of 55 studies. The full texts of the remaining 91 studies were thoroughly assessed, resulting in the final inclusion of 15 studies.^16-30^

### Study objects

All included studies were conducted by Chinese research teams (Table I). Seven studies adopted the discovery-validation two-step design, seven studies were single-cohort validation studies, and one study was a purely bioinformatics-based screening study without clinical cohort validation.

**Table I.** Experimental design of included studies.

Prediction role*	Country	Author (year)	Discovery		Validation	ARCO	Sample type	Methods	Biomarkers	AUC (95% CI)
			Study subjects	Data source						
**Diagnosis**	China	Mao et al^30^ (2025)	SONFH (n = 30) vs non-SONFH (n = 10)	GSE123568 GeneCards database	GSE74089: ONFH (n = 4) vs Fracture (n = 4) Recruitment: SONFH (n = 9) vs Healthy adults (n = 10)	I - IV	Peripheral blood, Hip articular cartilage	WGCNA, Machine learning, Immune cell infiltration analysis RT-qPCR	LCP1 ↑ PNP ↓ UBE2V1 ↓ ZFP36 ↑	0.92 (0.77 to 1.00) 0.95 (0.85 to 1.00) 0.97 (0.92 to 1.00) 0.94 (0.84 to 1.00)
	China	Zhao et al^29^ (2025)	NONFH (n = 64) vs Healthy adults (n = 64)	Hospital recruitment	None	I - IV	Peripheral blood	ECLI	β-CTx ↑ N-MID ↑	0.88 (0.82 to 0.94) 0.86 (0.80 to 0.92)
	China	Luo et al^27^ (2024)	SONFH (n = 30) vs non-SONFH (n = 10)	GSE123568	GSE74089: ONFH (n = 4) vs Fracture (n = 4) SONFH rats model vs Control model	I - IV	Peripheral blood, Hip articular cartilage femoral head tissues	WGCNA, PPI network, LASSO regression IF, WB, RT-qPCR	ICAM1 ↑ NR3C1 ↑ IKBKB ↑	Internal validation: 0.89 (0.73 to 0.99) External validation 1.00 (1.00 to 1.00)
	China	Wu et al^26^ (2024)	SONFH (n = 30) vs non-SONFH (n = 10)	GSE123568 GeneCards database	GSE74089: ONFH (n = 4) vs Fracture (n = 4) Recruitment: SONFH (n = 3) vs Healthy adults (n = 3)	I - IV	Peripheral serum, Hip articular cartilage	GSEA, PPI network, mRNA-miRNA network, CIBERSORT algorithm, RT-qPCR	CXCL8 ↑ STAT3 ↑ IL1B ↑ TLR4 ↑ PTGS2 ↑ TLR2 ↑ CASP1 ↑ CYBB ↑ HMOX1 ↑ CAT ↓	0.76 0.82 0.72 0.74 0.81 0.74 0.75 0.69 0.78 0.75 (95% CI: NR)
	China	Chai et al^25^ (2023)	SONFH (n = 30) vs non-SONFH (n = 10)	GSE123568	None	I - IV	Peripheral blood	GeneMANIA, PPI network, Correlation coefficient	NLRP1 ↑ NLRP3 ↑ CASP4 ↑ CASP1 ↑ CASP5 ↑ PYCARD ↑ SCAF11 ↑ NOD2 ↑ ELANE ↓ IL1B ↑	0.95 (0.87 to 1.00) 0.90 (0.77 to 1.00) 0.83 (0.67 to 0.99) 0.83 (0.66 to 1.00) 0.80 (0.64 to 0.96) 0.79 (0.59 to 1.00) 0.79 (0.57 to 1.00) 0.76 (0.58 to 0.95) 0.73 (0.51 to 0.95) 0.72 (0.48 to 0.96)
	China	Li et al^16^ (2019)	SONFH (n = 30) vs non-SONFH (n = 10)	Hospital recruitment	SONFH (n = 30) vs non-SONFH (n = 10)	I - IV	Peripheral blood	Microarray analysis, STRING network, partial least squares regression, qPCR	BIRC3 ↑ CBL ↑ CCR5 ↑ LYN ↑ PAK1 ↑ PTEN ↑ RAF1 ↑ TLR4 ↑	0.70 (0.54 to 0.84) 0.54 (0.37 to 0.70) 0.64 (0.48 to 0.79) 0.74 (0.58 to 0.87) 0.78 (0.63 to 0.90) 0.66 (0.50 to 0.81) 0.70 (0.53 to 0.83) 0.71 (0.54 to 0.84)
	China	Hong et al^17^ (2019)	AONFH (n = 5) vs Healthy adults (n = 5)	Hospital recruitment	AONFH (n = 30) vs Fracture (n = 30)	II - III	Peripheral blood, Femoral head specimens	microRNA PCR Panel, database bioinformatics, RT-qPCR	miR-1 ↓ miR-127-3 p ↓ miR-483-5 p ↑ miR-483-3 p ↑ miR-628-3 p ↓ miR-885-5 p ↑	0.90 (0.74 to 0.98) 0.97 (0.83 to 1.00) 0.90 (0.74 to 0.98) 0.96 (0.82 to 1.00) 0.96 (0.82 to 1.00) 0.93 (0.78 to 0.99)
**Progression**	China	Hou et al^28^ (2024)	SONFH (n = 36) vs Healthy adults (n = 36)	Hospital recruitment	None	II - IV	Peripheral blood	IHC, IF, ELISA Micro CT	p62 ↑	0.90 (95% CI: NR)
	China	Jia et al^24^ (2023)	SONFH (n = 30) vs non-SONFH (n = 10)	GSE123568 HPO database DisGeNET SoFDA	SONFH (n = 54) vs Healthy adults (n = 10)	I - IV	Peripheral blood	STRING network, functional interaction network, Co-expression network, RT-qPCR	PRKCA ↑ PIK3CD ↑ CD4 ↑ STAT2 ↑ CXCR4 ↑ LPAR1 ↑	Early-stage vs NC group: 0.89 Early-stage vs N&S group: 0.71 Early-stage vs NC group: 0.75 Early-stage vs N&S group: 0.78 Early-stage vs NC group: 1.00 Early-stage vs N&S group: 0.87 Late-stage vs NC group: 0.85 Late-stage vs N&S group: 0.71 Late-stage vs NC group: 0.80 Late-stage vs N&S group: 0.70 Late-stage vs NC group: 0.82 Late-stage vs N&S group: 0.92 (95% CI: NR)
	China	Peng et al^23^ (2023)	SONFH (n = 33) vs Healthy adults (n = 26)	Hospital recruitment	None	II - IV	Peripheral blood, Femoral head specimens	IHC, IF, ELISA	8-OHdG ↑	0.83 (95% CI: NR)
	China	Xiao et al^22^ (2023)	SONFH (n = 33) vs Healthy adults (n = 28)	Hospital recruitment	None	II - IV	Peripheral blood, Femoral head specimens	ELISA, IHC	4-HNE ↑	0.88 (95% CI: NR)
	China	Zheng et al^20^ (2022)	NONFH (n = 182) vs Healthy adults (n = 182)	Hospital recruitment	None	II - IV	Peripheral blood, Femoral head specimens	ELISA, RT-PCR, WB, IHC	CXCL12 ↓	0.62 (0.62 to 0.72)
	China	Wang et al^21^ (2022)	NONFH (n = 113) vs Healthy adults (n = 81)	Hospital recruitment	None	I - IV	Peripheral blood	ELISA	NAMPT ↓	Control vs NONFH: 0.81 ARCO2 vs ARCO3: 0.76 ARCO3 vs ARCO4: 0.67
	China	Liu et al^18^ (2020)	SONFH (n = 32) vs Healthy adults (n = 24)	Hospital recruitment	SONFH (n = 45) vs Healthy adults (n = 15)	II - IV	Peripheral blood	MB-WCX, MALDI-TOF-MS LC-ESI-MS/MS, ELISA, WB	FGA ↑	0.95 (95% CI: NR)
	China	Jiang et al^19^ (2020)	NONFH (n = 99) vs Healthy adults (n = 99)	Hospital recruitment	None	I - IV	Peripheral blood	RT-qPCR	CircCDR_1_as ↑	ARCO 1/2 vs 3: 0.70 ARCO 3 vs 4: 0.64 (95% CI: NR)

* “Diagnostic” indicates the biomarker was evaluated for its ability to distinguish NONFH patients from controls; “Progression” indicates the biomarker was associated with disease stage or severity.

AUC, area under the curve; ECLI, electrochemiluminescence immunoassay; ELISA, enzyme-linked immunosorbent assay; GSEA, gene set enrichment analysis; IF, immunofluorescence; IHC, immunohistochemistry; NC group, non-NONFH control group; NONFH, non-traumatic osteonecrosis of the femoral head; NR, not reported; N&S group, non-NONFH & other stage group; PPI, protein-protein interaction; RT-qPCR, reverse transcription quantitative polymerase chain reaction; SONFH, steroid-induced osteonecrosis of the femoral head; WB, Western blot; WGCNA, weighted gene coexpression network analysis.

Among the discovery sets, five studies had dataset overlap,^24-27,30^ all based on the GEO GSE123568 dataset. GSE123568 originated from the study by Li et al,^16^ in which the experimental group consisted of SONFH patients and the control group collected patients who received steroid administration against haematological diseases but did not develop SONFH. Three studies simultaneously integrated multiple public databases to supplement discovery set data.^24,26,30^ One study enrolled AIONFH patients and healthy normal controls.^17^

For the validation sets, all seven two-stage studies claimed to have used independent external validation cohorts. Among them, the study by Li et al^16^ adopted the most rigorous validation strategy, performing both internal ten-fold cross-validation and external independent validation. The GSE74089 dataset was the most frequently reused external validation set, in which the experimental group comprised patients with ONFH and the control group consisted of patients with femoral neck fracture without other diseases.^26,27,30^ Furthermore, some studies enrolled patients with SONFH or AIONFH along with healthy normal controls as independent validation cohorts, but it is noteworthy that the sample sizes of these validation cohorts were extremely small.^16,17,24,26,30^ One study validated biomarkers using a SONFH rat model in addition to the GSE74089 dataset.^27^

As for the single-cohort validation studies, the experimental group consisted of patients with SONFH or NONFH, while the control group comprised healthy normal subjects. Regarding NONFH staging, the subjects in most studies covered ARCO stages I to IV, while one study exclusively enrolled patients with ARCO stages II to III.^17^

### Analysis and experimental methods

All studies collected peripheral blood samples from participants. In the GSE74089 dataset, the sample type was hip articular cartilage. Additionally, four studies collected human femoral head tissues,^17,20,22,23^ and one study performed validation experiments using femoral head tissues from Sprague-Dawley rats.^27^

Several studies adopted multiomics integration strategies and employed network analysis to identify potential biomarkers. Following the acquisition of target datasets, weighted gene coexpression network analysis (WGCNA),^27,30^ gene set enrichment analysis (GSEA),^26^ and STRING-based protein-protein interaction (PPI) network construction were used.^16,24^ These approaches were combined with machine-learning algorithms and regression analysis to screen and prioritize candidate biomarkers.^27,30^ Beyond transcriptomics, one study identified differentially expressed proteins using MALDI-TOF-MS combined with LC-ESI-MS/MS,^18^ while another quantified serum biomarkers by electrochemiluminescence immunoassay (ECLI).^29^ In contrast, the remaining studies extracted potential biomarkers from existing literature and subsequently performed experimental validation at both gene and protein levels.

Reverse transcription quantitative polymerase chain reaction (RT-qPCR) was the most frequently used technique for nucleic acid-level validation, which the majority of studies further corroborated protein expression using Western blot (WB) or enzyme-linked immunosorbent assay (ELISA). Additionally, five studies conducted immunohistochemistry (IHC) or immunofluorescence (IF) assays on human or animal femoral head specimens.^20,22,23,27,28^ The predictive performance of candidate biomarkers or biomarker panels was evaluated by plotting receiver operating characteristic (ROC) curves and calculating the area under the curve (AUC). All biomarkers reported demonstrated favourable discriminative ability. Notably, the model of ICAM1, NR3C1, and IKBKB showed an AUC of 1.00.^27^ Different control groups yielded different predictive performances for CD4, when using non-NONFH as the control group, the AUC was 0.8; however, when using an overall control group comprising healthy adults and NONFH patients at middle/late-stages, the AUC reached 1.00.^24^

### Potential biomarkers

A total of 52 biomarkers were identified. Five studies reported gene-level biomarkers,^16,24-26,30^ two studies identified RNA-based biomarker,^17,19^ and the remainder at the protein level. Overall, in terms of functional application, eight studies primarily evaluated biomarkers in relation to disease progression, whereas seven studies focused on diagnostic purposes. Table II presents the GRADE evidence certainty for biomarkers that were either repeatedly identified or demonstrated favourable predictive performance. Detailed GRADE assessment results are provided in the Supplementary Appendix.

**Table II.** GRADE diagnostic accuracy.

Biomarker	Study number	Risk of bias	Indirectness	Inconsistency	Imprecision	Publication bias	Certainty of evidence	Potential role
IL1B	2	◉	◉	◯	◯	Strongly suspected	⨁◯◯◯	Diagnosis
TLR4	2	◉	◉	◯	◯	Strongly suspected	⨁◯◯◯	Diagnosis
CASP1	2	◉	◉	◯	◯	Strongly suspected	⨁◯◯◯	Diagnosis
ICAM1/NR3C1/IKBKB	1	◯	◉	◯	◉	Strongly suspected	⨁◯◯◯	Diagnosis
CD4	1	◯	◉	◯	◉	Strongly suspected	⨁◯◯◯	Progression
FGA	1	◯	◉	◯	◯	Strongly suspected	⨁⨁◯◯	Progression
CircCDR1as	1	◉	◉	◯	◯	Strongly suspected	⨁◯◯◯	Progression

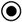
 = Serious; 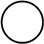 = Not serious; 
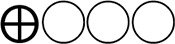
 = Very low; 
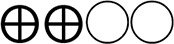
 = Low.

Risk of bias: Downgraded due to potential bias in lack of blinded external validation.

Indirectness: Downgraded because all included studies were exclusively conducted in Chinese populations, limiting generalizability to other ethnicities.

Imprecision: Downgraded for models relying on validation cohorts with exceptionally small sample sizes, which increases the risk of overfitting.

Publication bias: Strongly suspected due to the inherent nature of reporting positive findings in bioinformatics data mining.

CASP1, caspase 1; CD4, cluster of differentiation 4; FGA, fibrinogen alpha; ICAM1, intercellular adhesion molecule 1; IKBKB, inhibitor of nuclear factor kappa-B kinase subunit beta; IL1B, interleukin 1 beta; NR3C1, nuclear receptor subfamily 3 group C member 1; TLR4, toll-like receptor 4.

**Fig. 3:**
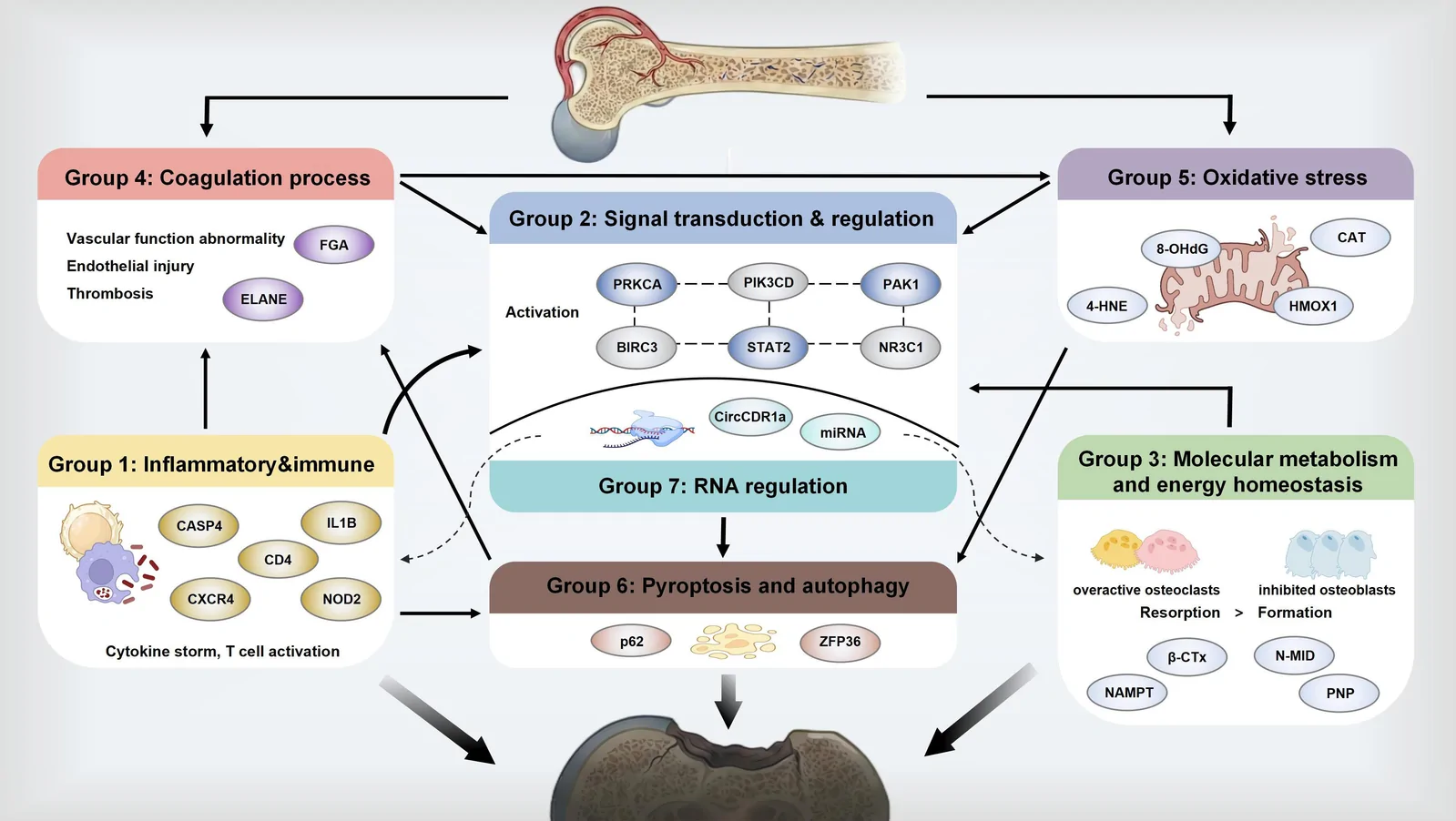
Schematic illustration of the potential molecular cascade mechanism and functional interaction network of non-traumatic osteonecrosis of the femoral head biomarkers.The diagram illustrates the hypothesized pathological progression from the initial microenvironmental insult of the femoral head to the final outcome of osteonecrosis, highlighting the potential functions of the seven biomarker groups. Solid arrows indicate hypothesized activation or progression, while dashed arrows represent potential regulatory function. Created with BioRender.com (BioRender, Canada). 4-HNE, 4-hydroxynonenal; CASP4, caspase 4; CD4, cluster of differentiation 4; CXCR4, C-X-C motif chemokine receptor 4; FGA, fibrinogen alpha; HMOX1, heme oxygenase 1; IL1B, interleukin 1 beta; miRNA, microRNA; NAMPT, nicotinamide phosphoribosyltransferase; NOD2, nucleotide-binding oligomerization domain-containing protein 2; NR3C1, nuclear receptor subfamily 3 group C member 1; PAK1, p21-activated kinase 1; PNP, purine nucleoside phosphorylase; PRKCA, protein kinase C alpha; STAT2, signal transducer and activator of transcription 2; ZFP36, zinc finger protein 36.

**Fig. 4:**
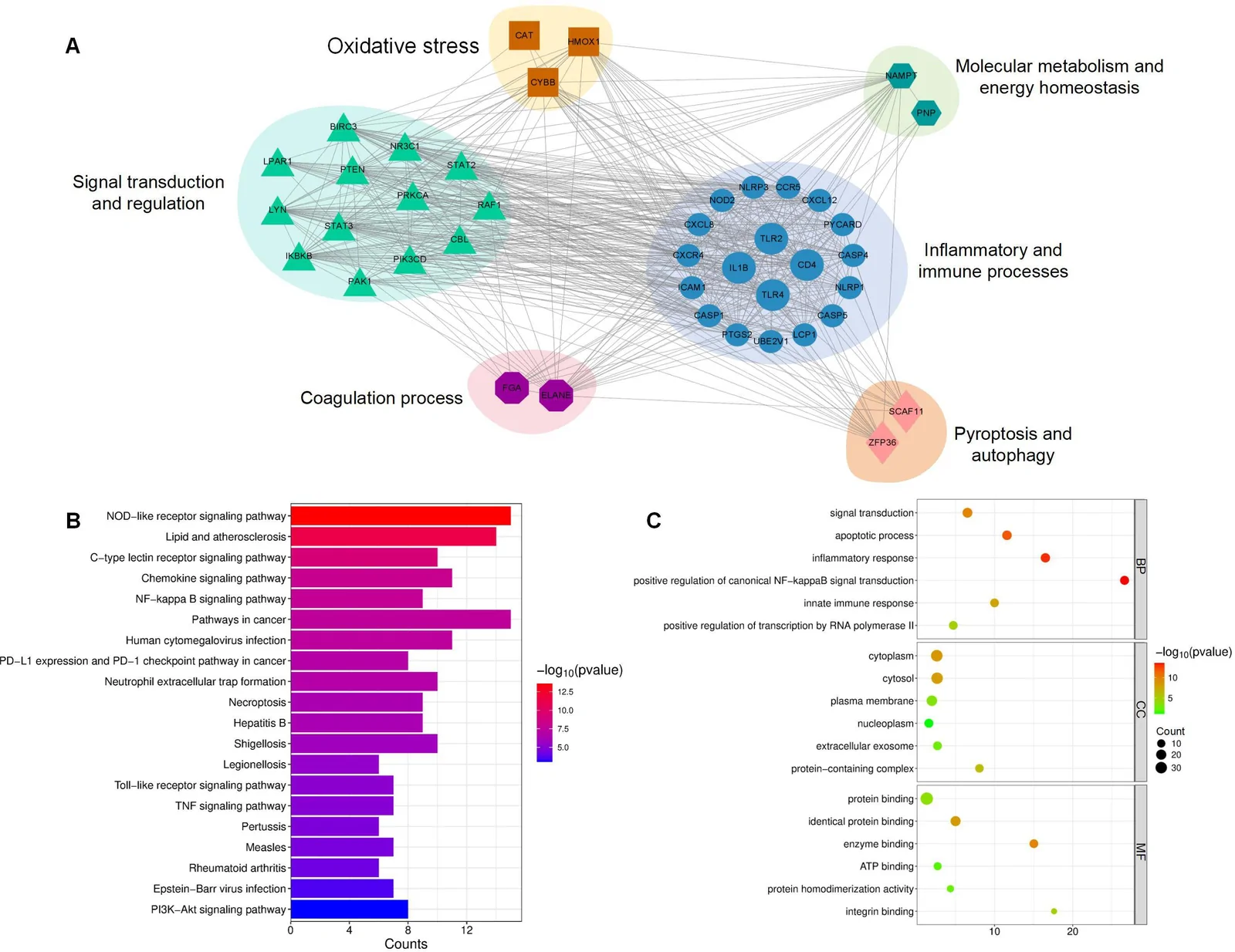
Function enrichment analysis of non-traumatic osteonecrosis of the femoral head (NONFH) biomarkers. a) Functional association network of NONFH biomarkers. b) Kyoto Enclyclopedia of Genes and Genomes pathway annotation. c) Gene Ontology biological processes. ATP, adenosine triphosphate; NF, nuclear factor; NOD, nucleotide-binding oligomerization domain; PD, programmed death; TNF, tumour necrosis factor.

As shown in Table III and Figure 3, the identified biomarkers were categorized into seven functional groups according to their core biological roles and associated pathways. A potential cascade interaction network was inferred from bioinformatics enrichment and observational data. Their expression patterns were further integrated to infer whether they exert predominantly promotive, protective, or dual effects in the pathological process of NONFH. Figure 4 illustrates the potential associations of these biomarkers in protein function and signalling pathways through KEGG pathway and GO analyses.

**Table III.** Summary of biomarker functions in non-traumatic osteonecrosis of the femoral head (NONFH).

Group and biomarker	Main functional	Association with NONFH	Expression trend
**Group 1: inflammatory and immune processes**			
CD4	Marker for T-helper cells, central to adaptive immune response	Promotion	↑
ICAM1	Mediates leucocyte adhesion and transmigration across endothelium	Promotion	↑
IL1B	Pro-inflammatory cytokine; activator of osteoclastogenesis	Promotion	↑
TLR2	Recognizes bacterial lipoproteins and DAMPs, amplifying inflammatory response	Promotion	↑
TLR4	Recognizes LPS and DAMPs, initiating NF-κB-driven inflammation	Promotion	↑
CASP1	Executes inflammasome-mediated pyroptosis and cleaves pro-IL1B	Promotion	↑
CASP4/5	Mediates non-canonical inflammasome pathway and pyroptosis	Promotion	↑
PTGS2	Key enzyme in prostaglandin synthesis, mediating pain, inflammation, and vascular permeability	Promotion	↑
PYCARD	Adaptor protein for inflammasome assembly	Promotion	↑
CXCL8	Potent chemokine for neutrophil recruitment and angiogenesis	Promotion	↑
CXCR4	Receptor for CXCL12, involved in homing of immune and stem cells	Promotion	↑
CCR5	Receptor for chemokines, recruiting monocytes/T-cells to inflammatory sites	Promotion	↑
LCP1	Participate in regulating immune responses; actin-binding protein	Promotion	↑
NOD2	Activates NF-κB and MAPK pathways	Promotion	↑
NLRP3	Forms inflammasome complex, leading to subsequent pyroptosis	Promotion	↑
NLRP1	Acts as the sensor component of the NLRP1 inflammasome, leading to subsequent pyroptosis	Promotion	↑
CXCL12	Chemokine regulating haematopoietic stem cell homing, leucocyte recruitment, and angiogenesis	Dual role	↓
UBE2V1	Involved in NF-κB activation and DNA repair	Dual role	↓
**Group 2: signal transduction and regulation molecules**			
IKBKB	Critical kinase for NF-κB activation	Promotion	↑
STAT3	Signal transducer and transcription activator	Promotion	↑
PTEN	Lipid phosphatase that antagonizes PI3K/Akt pathway	Dual role	↑
NR3C1	Glucocorticoid receptor	Dual role	↑
LPAR1	Receptor for LPA; regulates endothelial barrier function, cell migration, and osteoclastogenesis	Promotion	↑
BIRC3	Regulates cell apoptosis	Dual role	↑
PRKCA	Serine/threonine kinase involved in VEGF signalling, endothelial cell function, and osteoclastogenesis	Promotion	↑
PIK3CD	Catalytic subunit of PI3K, crucial for immune cell activation, migration, and survival	Promotion	↑
STAT2	Transcription factor activated by type I interferons, mediating antiviral response	Dual role	↑
LYN	Src-family kinase, regulates signalling in immune cells, osteoclasts, and endothelial cells	Dual role	↑
PAK1	Regulates cytoskeleton dynamics, cell motility, and osteoclast podosome formation	Promotion	↑
RAF1	Serine/threonine kinase in MAPK/ERK pathway, regulating cell growth and survival	Promotion	↑
CBL	E3 ubiquitin ligase that negatively regulates signalling from multiple receptors	Promotion	↑
**Group 3: molecular metabolism and energy homeostasis**			
PNP	Enzyme in catalyzing the phosphorolysis of purine nucleosides	Dual role	↓
β-CTx	Reflects bone resorption	Promotion	↑
N-MID	Reflects osteoblast activity and bone formation	Promotion	↑
NAMPT	Marker for osteogenic differentiation of BMSCs	Protection	↓
**Group 4: coagulation process**			
ELANE	Pyroptosis; promotes blood coagulation	Protection	↓
FGA	Central component of blood clots; implicated in intravascular coagulation and ischaemia	Promotion	↑
**Group 5: oxidative stress**			
HMOX1	Catalyzes the oxidative cleavage of heme; affords protection against programmed cell death	Dual role	↑
CAT	Catalase that defends against oxidative stress	Dual role	↓
CYBB	Catalytic subunit of NADPH oxidase, responsible for ROS production in phagocytes	Dual role	↑
8-OHdG	Oxidized guanosine nucleoside	Promotion	↑
4-HNE	Reactive aldehyde generated from lipid peroxidation	Promotion	↑
**Group 6: pyroptosis and autophagy**			
ZFP36	RNA-binding protein that destabilizes mRNAs of inflammatory cytokines	Protection	↑
p62	Adaptor protein at the nexus of autophagy, oxidative stress response, and cell signalling	Dual role	↑
SCAF11	Involved in transcription termination and RNA processing	Dual role	↑
**Group 7: RNA regulation**			
CircCDR1as	Circular RNA acting as a molecular sponge for miRNAs, influencing gene expression networks	Promotion	↑
miR-1	Osteoblast, osteoclast and endothelial cell development; regulate the expression of mRNA in the post-transcription process by inducing the RNA silencing complex	Protection	↓
miR-127-3p		Protection	↓
miR-483-5p		Promotion	↑
miR-483-3p		Promotion	↑
miR-628-3p		Protection	↓
miR-885-5p		Dual role	↑

BMSCs, bone marrow mesenchymal stem cells; CASP, caspase; CD4, cluster of differentiation 4; CXCL, C-X-C motif chemokine ligand; DAMPs, damage-associated molecular patterns; FGA, fibrinogen alpha; HMOX1, heme oxygenase 1; 4-HNE, 4-hydroxynonenal; ICAM1, intercellular adhesion molecule 1; IKBKB, inhibitor of nuclear factor kappa-B kinase subunit beta; IL1B, interleukin 1 beta; LCP1, lymphocyte cytosolic protein 1; LPA, lysophosphatidic acid; LPAR1, lysophosphatidic acid receptor 1; LPS, lipopolysaccharide; MAPK, mitogen-activated protein kinase; miRNA, microRNA; NAMPT, nicotinamide phosphoribosyltransferase; NAPDH, nicotinamide adenine dinucleotide phosphate; NF-κB, nuclear factor kappa-light-chain-enhancer of activated B cells; NOD2, nucleotide-binding oligomerization domain-containing protein 2; NR3C1, nuclear receptor subfamily 3 group C member 1; 8-OHdG, 8-hydroxy-2’-deoxyguanosine; PAK1, p21-activated kinase 1; PNP, purine nucleoside phosphorylase; PRKCA, protein kinase C alpha; PTEN, phosphatase and tensin homolog deleted on chromosome 10; PTGS2, prostaglandin-endoperoxide synthase 2; ROS, reactive oxygen species; STAT3, signal transducer and activator of transcription 3; TLR, toll-like receptor; UBE2V1, ubiquitin-conjugating enzyme E2 variant 1; VEGF, vascular endothelial growth factor; ZFP36, zinc finger protein 36.

### Group 1: Immune-inflammatory activation and response

This group includes CXCL12,^20^ CCR5,^16^ TLR4,^16,26^ TLR2, CXCL8, PTGS2,^26^ CXCR4, CD4,^24^ IL1B,^25,26^ ICAM1,^27^ NOD2, CASP1/4/5, PYCARD,^25^ LCP1, and UBE2V1.^30^ Except for UBE2V1 and CXCL12, all biomarkers in this group were markedly upregulated, collectively suggesting a predominantly promotive role in the pathogenesis of NONFH. The downregulation of UBE2V1 and CXCL12 indicated that they may exert context-dependent, bidirectional regulatory effects on inflammation and the local microenvironment.

### Group 2: Signal transduction and regulation

This group mainly comprises IKBKB,^27^ STAT3,^26^ STAT2, LPAR1, PRKCA, PIK3CD,^24^ CBL, PTEN, PAK1, LYN, RAF1, BIRC3,^16^ and NR3C1.^25^ These molecules exhibited an overall up-regulation trend, implying that they contribute to the promotive or amplifying effect in NONFH pathogenesis. NR3C1, BIRC3, STAT2, and LYN may also participate in negative feedback or protective action, thereby displaying potential dual regulatory characteristics.

### Group 3: Molecular metabolism and energy homeostasis

Upregulation of β-C-terminal telopeptide of type 1 collagen (β-CTx) and N-terminal midfragment of osteocalcin (N-MID) reflected enhanced bone resorption and remodeling activity, indicating an imbalance in bone metabolism.^29^ Beyond its role in bone anabolism, NAMPT also participates in lipid metabolism. The significant downregulation observed across ARCO stages (2 vs 3 vs 4) indicates its potential as a biomarker for monitoring NONFH progression.^21^ Although PNP also showed a decreasing trend, its multiple roles in purine metabolism and immune regulation suggest a possible bidirectional influence.^30^

### Group 4: Vascular function and coagulation

Fibrinogen α-chain (FGA) was upregulated, favoring microthrombus formation and thereby promoting the progression of NONFH.^18^ In contrast, ELANE expression was downregulated, which may attenuate neutrophil elastase-mediated tissue damage and thus confer a certain degree of protection.^25^

### Group 5: Oxidative stress

Consistent upregulation of HMOX1, CYBB,^26^ 4-hydroxynonenal (4-HNE),^22^ and 8-hydroxy-2’-deoxyguanosine (8-OHdG),^23^ coupled with downregulation of CAT,^26^ indicates an elevated state of oxidative stress accompanied by increased DNA and lipid damage, collectively contributing to NONFH progression. Notably, cross-validation with clinical samples revealed a divergent expression pattern for HMOX1, CYBB, and CAT compared to the GSE74089 dataset. In clinical specimens, HMOX1 and CYBB were significantly downregulated, whereas CAT was markedly upregulated, suggesting context-dependent or bidirectional regulatory functions for these molecules.^26^

### Group 6: Pyroptosis and autophagy

SCAF11, NLRP1, NLRP3,^25^ ZFP36,^31^ and p62^28^ were all upregulated, suggesting that inflammasome activation, pyroptotic cell death, and autophagy-related pathways are engaged in the pathological process of NONFH. At the same time, p62 and SCAF11 may possess both pro-inflammatory and protective potential.

### Group 7: RNA regulatory

CircCDR1as,^19^ miR-483-5p, miR-483-3p, and miR-885-5p were upregulated, whereas miR-1, miR-127-3p, and miR-628-3p^17^ were downregulated. These alterations suggested the existence of a miRNA-centered regulatory network in NONFH.

## Discussion

The reliance on imaging methods often leads to a delayed diagnosis of NONFH. This systematic review aimed to synthesize the most recent evidence on biomarkers for the diagnosis and progression prediction of NONFH, and summarizes the methodologies and key outcomes, including biomarker expression trends, predictive performance, and their potential biological roles. Functional annotation affirms that these biomarkers may collectively contribute to the pathological processes of bone metabolism dysregulation and vascular dysfunction in NONFH by mediating mechanisms such as inflammatory-immune imbalance, cellular metabolic disturbances, and abnormal nucleic acid level. Our network intentionally emphasizes that blood-based biomarkers can serve as a rapid and easily accessible diagnostic tool, providing a critical timeframe for hip-preserving treatment before the onset of mechanical collapse.

We found that these studies generally lack the raw data needed to construct diagnostic fourfold tables methodologically, and some studies did not fully report 95% CIs. It is impossible for this review to perform quantitative meta-analysis or sensitivity analysis based on statistical software. Furthermore, significant clinical and methodological heterogeneity existed across studies in terms of detection platforms, biomarker cut-off values, and control group definitions (e.g. healthy adults vs femoral neck fracture patients), making quantitative synthesis of heterogeneity statistically inappropriate.

To address these challenges and ensure transparency, we conducted a qualitative sensitivity analysis instead of a quantitative one. Our comparative analysis revealed that despite the risk of bias from dataset overlap, the predictive performance of core biomarkers (specifically IL1B, TLR4, and CASP1) remained highly consistent between independent clinical validation cohorts and findings derived from public database mining. Within the GRADE framework, this consistency in both the direction and magnitude of the effect supports the robustness of our findings. Nevertheless, given that current evidence is predominantly associative rather than indicative of a direct causal mechanism, the diagnostic performance estimated in this review should be regarded as exploratory. Future multicentre, large-scale prospective independent studies are imperative to validate their clinical predictive utility.

Current evidence indicates that the core pathological process of NONFH is osteocyte necrosis secondary to disruption of the femoral head blood supply, accompanied by an imbalance between osteoblastic and osteoclastic activity.^6^ β-CTx is a key biomarker of bone resorption.^32^ N-MID, synthesized by osteoblasts, is widely used as a serum marker of bone formation.^33^ Zhao et al^29^ reported that serum β-CTx and N-MID levels are significantly elevated in NONFH patients and positively correlated with each other, suggesting an excessively activated bone remodelling state and can be used for early prediction of NONFH.

Microcirculatory disturbance caused by intravascular microthrombosis further aggravates ischaemic necrosis of bone cells.^34^ Liu et al^18^ demonstrated that FGA is markedly upregulated in the peripheral blood of SONFH patients. Beyond promoting coagulation, FGA may also influence local inflammatory responses by regulating fibrin cross-linking. In contrast, Chai et al^25^ observed downregulated expression of ELANE, a molecule involved not only in pyroptosis but also in platelet aggregation and coagulation processes.^35^ These findings collectively suggest the necessity of considering vascular dysfunction-related indicators as candidate biomarkers for NONFH.

The mechanisms by which oxidative damage contributes to NONFH may include elevated levels of reactive oxygen species (ROS) promoting osteoclast differentiation, inducing apoptosis in osteoblasts and osteocytes, and sustained high oxidative stress leading to decreased expression of antioxidant enzymes, ultimately resulting in excessive bone resorption.^36^ Studies by Wu et al,^26^ Peng et al,^23^ and Xiao et al^22^ have shown that multiple oxidative stress-related products are significantly elevated in peripheral blood. CYBB is responsible for ROS generation, HMOX1 participates in regulated cell death, 8-OHdG is produced from oxidized guanine in damaged DNA, and 4-HNE is one of the major bioactive end products of lipid peroxidation of polyunsaturated fatty acids. Additionally, Wu et al^26^ reported decreased expression of CAT, a key enzyme in counteracting oxidative stress. Collectively, these biomarker profiles indicate excessive oxidative stress within the femoral head, and oxidative stress-related biomarkers may serve as early warning indicators of NONFH.

This systematic review suggests that dysregulated immune-inflammatory activation may represent a central pathological event in the early stages of NONFH. CD4, a surface marker of T helper cells, serves as a core molecule for the initiation and regulation of adaptive immune responses.^37^ Pattern recognition receptors, including TLR4 (which recognizes lipopolysaccharides and damage-associated molecular patterns, DAMPs) and TLR2 (which senses bacterial lipoproteins and DAMPs), act as ‘immune sentinels’ to initiate innate immune responses. NOD2 is an intracellular pattern-recognition receptor that senses cytosolic pathogen-associated components and activates the NF-κB pathway.^38^ NLRP1/NLRP3 forms inflammasome complexes that trigger CASP1-mediated pyroptosis and IL-1β-driven local inflammatory responses.^39^ CASP4 and CASP5 are key executors of the non-canonical inflammasome pathway, induced to trigger pyroptosis under the regulation of NF-κB.^40^ PYCARD functions as an adaptor protein within inflammasomes and is a critical bridge through which NLRP3 or CASP1 induce inflammation and pyroptosis.^41^ PTGS2 encodes inducible cyclooxygenase-2 (COX-2), which produces prostaglandins in response to inflammatory stimuli and mediates fever, pain, and vascular responses.^42^

A network of chemokines and adhesion molecules orchestrates the recruitment and infiltration of immune cells into the femoral head during NONFH progression. The potent chemokine CXCL8 plays a central role in recruiting and activating neutrophils towards inflammation sites. CXCR4, upon binding its ligand CXCL12, mediates the homing and directional migration of lymphocytes and hematopoietic stem cells.^43^ LCP1 is a lymphocyte-specific actin-bundling protein that regulates the motility and migratory architecture of immune cells.^44^ CCR5, a CC-like chemokine G-protein-coupled receptor, guides the directional migration of immune cells associated with NF-κB, PI3K/Akt, and JAK-STAT signalling pathways.^45^ The vascular endothelial adhesion molecule ICAM1 facilitates firm adhesion and transendothelial migration of leucocytes through specific binding to integrins on the T cell surface.^46^

Notably, molecules such as IL1β and PTGS2 are not only effector molecules generated during ‘immune recognition and response’, but also strong inducers of ‘recruitment and infiltration’ molecules, including ICAM1 and CXCL8. In this way, they couple the recognition and trafficking phases into a coherent inflammatory positive-feedback loop. The observed downregulation of CXCL12 and UBE2V1 suggests that both may exert bidirectional regulatory roles. For example, CXCL12, under certain conditions, may promote stem cell retention and tissue repair, whereas the downregulation of UBE2V1 may be related to the gene expression of autophagy-related inhibitory genes.^47^

Our findings indicate that during the progression of osteonecrosis of the femoral head, cell death is not only regulated by classical protein signalling pathways but also profoundly influenced by a complex post-transcriptional regulatory network comprising circular RNAs, microRNAs, and RNA-binding proteins. Aberrant accumulation of the autophagy receptor and signalling scaffold protein p62 promotes apoptotic and necrotic cell death.^28^ Meanwhile, the RNA-binding proteins SCAF11 and ZFP36 participate in the fine-tuning of cell fate by regulating pre-mRNA splicing and mature mRNA stability, respectively.^48^

CircCDR1as is a highly conserved circular RNA whose canonical role involves acting as a ‘sponge’ for miR-7 and other miRNAs.^31^ The sequestered or freely circulating microRNAs serve as direct effector molecules, influencing the protein expression levels of key pro-apoptotic and anti-apoptotic genes by targeting specific ‘miRNA–target gene’ regulatory axes. This in turn affects various biological processes, including cell cycle regulation and programmed cell death. The multilayered mechanisms of this regulatory network provide new molecular insights into the processes of cell death in osteonecrosis of the femoral head. Research by Chen et al^49^ founded that exosomes inhibit the HIF-1α/VEGFA pathway by carrying miR-155-5 p, thereby impairing the function of vascular endothelial cells. Hong et al^17^ revealed that miRNAs influence genes associated with angiogenesis and osteogenesis functions, including VEGFA, PDGFA, and PTEN, indicating that miRNAs effectively regulate angiogenesis and osteogenesis at the post-transcriptional level. Research by Chen et al^50^ demonstrated that exosomes inhibit the HIF-1α/VEGFA pathway by carrying miR-155-5p, thereby impairing the function of vascular endothelial cells.

Integration of biomarker functions and associated signalling pathways indicates that NONFH may be driven by a complex pathological network characterized by multilevel and multipathway synergistic interactions. The key feature of this network lies in the fact that initial injury activates the innate immune recognition system, triggering a sustained and amplified inflammatory response. Subsequently, through complex signal transduction hubs, this response may contribute to bone metabolism imbalance, vascular dysfunction, and oxidative stress damage, ultimately leading to the death of osteoblasts and osteocytes as well as the collapse of bone tissue.

The pathological cascade may be initiated by the inflammation initiation.^51^ Under triggers such as ischaemia, high-dose glucocorticoid exposure, or metabolic derangements, necrotic osteocytes and damaged matrix release large amounts of DAMPs. These endogenous danger signals are sensed by pattern-recognition receptors such as Toll-like receptors (TLR2/4) and NOD-like receptors (NOD2). Upregulation of these receptors marks a state of heightened immune vigilance. Subsequently, they activate the downstream NF-κB pathway and raise kinase IKBKB level, driving the production of large quantities of inflammatory mediators such as IL-1B and PTGS2. Particularly critical is the activation of the NLRP3-centered inflammasome, which cooperates with CASP1/4/5 to directly trigger the inflammatory process of pyroptosis.

Once inflammation is initiated, it is sustained and amplified through chemokine signalling pathways. The highly expressed CXCL8, in synergy with the upregulated adhesion molecule such as ICAM1, efficiently recruit neutrophils and other inflammatory cells into bone tissue. Concurrent upregulation of CXCR4 and CCR5 promotes infiltration of monocytes/macrophages and lymphocytes. These recruited immune cells further release inflammatory mediators, creating a positive feedback loop and establishing a chronic inflammatory microenvironment that is difficult to resolve. Chronic inflammatory signals are then integrated and converted into cell fate decisions through a series of intracellular signal transduction hubs. Pro-survival/pro-proliferative signals, driven by molecules such as the PI3K-Akt pathway and RAF1-mediated proliferative/survival signalling, are abnormally enhanced, potentially reflecting compensatory responses to stress but also contributing to aberrant repair. Meanwhile, imbalance in key negative regulators such as PTEN results in inadequate restraint of these pathways. STAT3, as a central transcription factor, is persistently activated. It not only amplifies inflammation but also promotes osteoclast differentiation and suppresses osteoblast function, thereby directly serving as a bridge linking inflammation to bone destruction.

These disrupted inflammatory and signalling networks always contribute to critical pathological effector processes. First, vascular dysfunction ensues. Inflammatory mediators damage the endothelium, increased FGA levels indicate a local hypercoagulable state, and downregulation of protective factors such as ELANE compromises vascular stability. These changes aggravate microcirculatory disturbance and ischaemia, establishing a vicious cycle of ‘ischemia - inflammation - re-ischemia’. Second, dysregulated lipid metabolism represents a critical pathological component that cannot be overlooked. The activation of signalling pathways such as PI3K/Akt and NF-κB modulates lipid synthesis and suppresses lipolysis, primarily through mediating downstream insulin signalling.^52,53^ Activated inflammatory cells generate large amounts of ROS, resulting in accumulation of oxidative damage markers and thus pronounced oxidative stress. 4-HNE also serves as a direct indicator of the severity of lipid peroxidation damage.^54^ Additionally, NAMPT, functioning as an adipokine, participates in the regulation of lipid metabolism and insulin sensitivity. Its downregulation may exacerbate local lipid accumulation and adipocyte differentiation. This process can increase bone marrow fat volume and elevate intraosseous pressure, ultimately further compromising the blood supply to the femoral head.^55,56^ Third, the intense inflammatory milieu strongly stimulates osteoclast activation, which is reflected in the elevation of β-CTx as a bone resorption marker. Although the bone formation marker N-MID may also increase in a compensatory manner, indicating high bone turnover, sustained inflammatory injury ultimately impairs osteoblast function, leading to a breakdown of the dynamic balance between bone resorption and formation and a net loss of bone mass.

In addition, the reviewed studies reveal deeper regulatory mechanisms. Non-coding RNAs such as CircCDR1as participate in complex competing endogenous RNA networks by sequestering miRNAs, and may globally modulate the aforementioned signalling pathways at an epigenetic-like level.^57^

Several limitations of this systematic review should be acknowledged. First, all included studies were conducted within Chinese populations. This is primarily because studies from non-Asian regions were limited in number, and were excluded based on our rigorous PICOS criteria during the retrieval process. Given the potential ethnic variability in biomarker expression and genetic predispositions to NONFH, the external validity of these findings is limited. The applicability of this pathophysiological cascade network to Western or other ethnic cohorts requires further cross-population investigation.

Second, the majority of the reviewed studies lack high-quality, independent external clinical validation. Relying solely on internal clinical testing without cross-validation may lead to an over-optimistic estimation of a biomarker’s predictive performance. Furthermore, for studies heavily dependent on public datasets (such as GEO), the exceptionally small sample sizes in their validation cohorts considerably increase the risk of overfitting in the predictive models. Because the discovery sets of five studies originated from the same GSE123568 dataset reported by Li et al,^16^ these studies cannot be regarded as independent sources of evidence. Consequently, the evidence supporting the three replicated biomarkers – IL1B, TLR4, and CASP1 – is of relatively low strength.

Third, existing studies lack an in-depth exploration of the specific mechanisms by which these biomarkers participate in the pathogenesis of NONFH. The multidimensional pathophysiological cascade network proposed in this study is predominantly derived from observational clinical data and bioinformatics enrichment analyses, which provide associative rather than definitive causal evidence. Although these circulating biomarkers are statistically correlated with the onset or progression of NONFH, this does not directly equate to a deterministic driving role in its pathogenesis. Future rigorous in vivo and in vitro experimental studies (e.g., using knockout or overexpression models) are imperative to validate the precise biological functions and causal mechanisms of these core biomarkers.

In summary, biomarkers for NONFH exhibit considerable potential for early diagnosis and disease progression prediction in the Chinese population. NONFH does not result from a defect in a single pathway, but rather constitutes a cascade network characterized by interconnected and mutually reinforcing processes of ‘immune activation - chronic inflammation - signal integration - oxidative stress, lipid metabolism - microcirculatory disturbance - imbalance of bone metabolism’. This suggests that future therapeutic strategies should adopt multitarget, multi-pathway combinatorial interventions. Approaches such as blocking inflammasome activation, selectively inhibiting PI3Kδ, or targeting key circRNAs may hold promise for breaking this pathological vicious cycle, thereby opening new avenues for precision medicine in the management of NONFH.

## Data Availability

All data generated or analyzed during this study are included in the published article and/or in the supplementary material.
